# On the Front Lines of Lassa Fever

**DOI:** 10.3201/eid1010.IM1010

**Published:** 2004-10

**Authors:** Daniel G. Bausch, Sanie S.S. Sesay, Babafemi Oshin

**Affiliations:** * Tulane School of Public Health and Tropical Medicine, New Orleans, Louisiana, USA;; † Kenema Government Hospital, Kenema, Sierra Leone;; ‡ Merlin Sierra Leone, Freetown, Sierra Leone

**Keywords:** Aniru Conteh, Lassa Fever, Obituary

**Figure:**
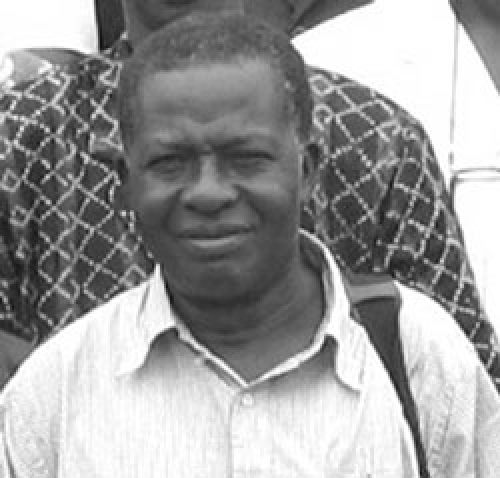
Photo by F. Jacquerioz

Aniru Conteh spent 25 years in his native Sierra Leone dedicated to treating patients with Lassa fever, a rodentborne viral disease, to which he ultimately succumbed on April 4, 2004. Dr. Conteh's life is a model of the dedicated healthcare worker. His colleagues hope that his death can galvanize support for healthcare workers and scientists working on the front lines with Lassa fever virus and other emerging pathogens.

The son of the local chief, Aniru Sahib Sahib Conteh was born in the small village of Jawi Folu in Eastern Province, Sierra Leone, in 1942. When Conteh was 16 years of age, his mother died, and he left school to help support the family in the capitol, Freetown. He eventually returned to school, where he studied chemistry and biology, and earned his bachelor's degree from Durham University, Freetown. After working briefly as a teacher, he enrolled in medical school at the University of Ibadan, Nigeria. He graduated in 1974 and stayed on to work at Ibadan Teaching Hospital. In 1979, Dr. Conteh returned to Sierra Leone, beginning what would be a 25-year career dedicated principally to the fight against Lassa fever.

Lassa fever was first recognized in 1969 after three nurses working at a mission hospital in North-Eastern State, Nigeria, came down with a mysterious illness (*1**,**2*). A new virus was subsequently isolated from a blood specimen sent to the Yale Arbovirus Research Unit and named Lassa after the village of origin of the first case-patient (*3*). A larger outbreak, 28 cases with at least 14 deaths, occurred in the same region in 1970 (*4*). Lassa fever was first identified in Sierra Leone in 1972 in a series of nosocomial outbreaks (*5**,**6*). The disease was found to be common in the community as well, constituting a major cause of illness and death in eastern Sierra Leone, which prompted the Centers for Disease Control (CDC) to establish a Lassa fever research and control program in Sierra Leone in 1976 (*7*).

Dr. Conteh willingly plunged into this hotbed of Lassa fever in 1979 when he took a post at Nixon Methodist Hospital in the eastern town of Segbwema, the central hospital of CDC's program. He was named Nixon Hospital's medical superintendent in 1980 and later served as the clinical director of the Lassa fever treatment ward. When civil war broke out in 1991, the treatment ward was moved to the relative safety of nearby Kenema Government Hospital, and Dr. Conteh continued as its director. The war eventually forced the CDC program to close, but Dr. Conteh and the Lassa fever ward carried on through the support of the British medical relief agency, Merlin.

From 1979 to 2004, Dr. Conteh treated thousands of patients with Lassa fever, becoming the unparalleled world's expert on the management of the disease, as well as contributing to research on the subject (*8*). He persevered despite many risks—outbreaks of Lassa fever, rebel invasions, and government counterattacks. Through various projects and experts, war and peace, and waves of refugees, Dr. Conteh stayed, continuing to treat patients in his characteristically calm and modest manner. His dedication, skill, and courage were some of the few constants in the unstable and often dangerous world around him.

In March 2004, Dr. Conteh admitted a young, pregnant woman to the Lassa ward with a presumptive diagnosis of severe Lassa fever. The patient was a volunteer nurse on the hospital's pediatric service. On March 17, after numerous unsuccessful attempts by staff members to obtain blood from the patient's arm, Dr. Conteh attempted femoral venipuncture and sustained a needlestick injury in the process. The patient died the next day. On March 23, fever developed in Dr. Conteh. Despite the administration of intravenous ribavirin, profuse vomiting and diarrhea developed a few days later; these led to hypovolemic shock and cardiac arrest, which necessitated resuscitation. Bleeding and renal failure ensued. Consultations were sought and received from medical experts around the world. The diagnosis of Lassa fever was confirmed from specimens sent to the National Institute for Communicable Diseases in South Africa. On April 4, in the cruelest irony, Aniru Conteh died of a virus that he had been combating as a physician for most of his life, a patient in a ward that he had been instrumental in establishing and maintaining.

Dr. Conteh's death represents more than a personal loss. His absence severely undermines the ability to combat Lassa fever, which remains a serious threat. Research over the years has shown that Lassa fever is endemic in Liberia, Guinea, Nigeria, and Senegal as well, and Lassa virus is now believed to infect tens of thousands of people and cause thousands of deaths yearly across West Africa (*7**,**9**–**13*).

What lessons can we learn? What response can we have to this tragedy? We have made progress in our global response to emerging pathogens, but many challenges remain. Perhaps the most important response is to offer a sound base of support for combating emerging diseases where they start, relying less on the rapid influx of international experts and the long-distance shipping of specimens and more on "home grown" talent, equipped with the tools and training that they need. Achieving these goals will be difficult. Beyond supplying medicine and laboratory equipment, tackling the problem will require addressing such complex issues as low salaries and "brain drain," civil unrest, corruption, and human rights. Dr. Conteh was exceptional because he persisted in the face of these challenges, but we cannot routinely depend on such heroes. Governments in developing countries, with international support, need to build the base to create stable job and training opportunities, adequate physical infrastructure, and safe working environments to foster the development of local expertise and encourage local physicians and scientists to help fill the role vacated by Dr. Conteh. Ultimately, containing emerging diseases depends on the Aniru Contehs of the world. The more support we provide to people on the front lines, the healthier and safer we all will be.

Aniru Conteh is survived by his wife, Sarah, three sons, and two daughters.
